# 

**Published:** 1908-08

**Authors:** 


### CONTENTS.

ORIGINAL COMMUNICATIONS:—

Sacro-Iliac Diseases. By C. R. Andrews, M. D., and Michael Hoke, M. D., Atlanta, Ga... 251

The Examination of the Foeces as an Aid to Diagnosis. Dr. Jos. Le Conte, Lecturer Diseases of the Stomach, Atlanta School of Medicine. Gastrologist to the Presbyterian Hospital, Atlanta, Ga. .. .. .. .. 250

The Use and Abuse of Drugs in Tuberculosis. By William M. Jones, M. D., High Point, N. C. .. .. .. .. 259

Chronic Stomach Ulcer; Its Surgical Treatment. By Edward G. Jones, A. B., M. D., Professor of Surgery in the Atlanta School of Medicine .. .. .. .. 264

Obstetrical Work from the Standpoint of the General Practitioner. By A. B. Croom, M. D., Maxton, N. C.... .. .. .. .. 272

Variable Effects of Apormorphine. Frank K. Roland, M. D., Atlanta, Ga .. .. .. .. 274

Changes of Date of the Atlanta Meeting of the Association of Military Surgeons .. .. .. 276

An Improvement in the Method of Determining if Gonorrhoeal Infection is preesnt in an Inflamed Prostate Gland or Seminal Vesicle. By Edgar Ballenger, M. D., Lecturer on Genito-Urinary Diseases, Atlanta Sschool of Medicine, Atlanta, Ga. .. .. .. .. 277

EDITORIALS .. .. .. .. 280

NEWS AND NOTES .. .. .. .. 286

BOOK REVIEWS .. .. .. .. 292

